# In your face: transcendence in embodied interaction

**DOI:** 10.3389/fnhum.2014.00495

**Published:** 2014-07-09

**Authors:** Shaun Gallagher

**Affiliations:** ^1^Department of Philosophy, Lillian and Morrie Moss Professor of Excellence in Philosophy, University of MemphisMemphis, TN, USA; ^2^Department of Philosophy, School of Humanities, University of HertfordshireHatfield, Hertfordshire, UK; ^3^Department of Philosophy, Faculty of Law, Humanities and the Arts, University of WollongongWollongong, NSW, Australia

**Keywords:** interaction, face, ethics, transcendence, Levinas

## Abstract

In cognitive psychology, studies concerning the face tend to focus on questions about face recognition, theory of mind (ToM) and empathy. Questions about the face, however, also fit into a very different set of issues that are central to ethics. Based especially on the work of Levinas, philosophers have come to see that reference to the face of another person can anchor conceptions of moral responsibility and ethical demand. Levinas points to a certain irreducibility and transcendence implicit in the face of the other. In this paper I argue that the notion of transcendence involved in this kind of analysis can be given a naturalistic interpretation by drawing on recent interactive approaches to social cognition found in developmental psychology, phenomenology, and the study of autism.

## Introduction

What I see in the other’s face is irreducible to its physiogomy, its shape or morphological features, its color or physical properties. The significance of the face transcends any of these things. This is an insight associated with the philosophy of Emmanuel Levinas (1969). The other person, in her otherness, resists being simply an entity—whether physical object or epistemological subject. The other is not the sum total of her ontological parts, but in some way transcends all of those parts. Nor is this transcendence simply a way of pointing to something invisible, a mind or a set of mental states that we might be able to infer or simulate. The other is not, analogically, another *me*, or a set of mental states that are like mine. Rather, Levinas suggests, the other, in her alterity, makes an ethical demand on me, to which I am obligated to respond—the face-to-face is primarily an ethical relation—the other’s face is perceived as an obligation to respond.

For Levinas, ethics is not a matter of theory, argumentation or the promulgation of rules, but is based on an experience of transcendence encountered in the other’s face. The situation in which I experience this transcendence is “when the face has turned to me, in its very nakedness. It is by itself, and not by reference to a system” (Levinas, 1969, p. 75). In this circumstance the other’s vulnerability shines through her face, independent of context, and elicits a response from me.

The face is characterized by proximity and distance at the same time. When the other’s face is close to me, it is so not merely in physical geographical terms, the way an instrument or artifact might be. Its closeness demands a response that could range from a passionate kiss to a punch, or some less extreme and more polite behavior of moving away or asking for space. Even in contexts that involve a close examination in scientific or medical terms, the face demands some form of respect. Yet, even in its closeness there is something distant in it since one’s experience of the other is not just in terms of the physicality of the face. The face (or more generally, the body) is never the totality of the other.

Although Levinas is in some respects a religious thinker (Veling, 1999; Purcell, 2006), his ethics is not necessarily religious, and his thinking about the face can be interpreted in secular terms of embodied, and especially affective, intersubjective experience. At least on one reading (Bergo, 2011), his ethical concept of transcendence is not informed by his religious thought; it is rather the other way around. Religious thinking may be motivated by the transcendence encountered in our intersubjective relations. Ethics begins in face-to-face experience and not in a theological dictum or reference to God. At the same time, however, Levinas (1991) associates the notion of transcendence indicated in the other’s face with a form of infinity and as something beyond the reach of science. It is something that is “beyond understanding” (déborde la compréhension) (Levinas, 1991, p. 18).

What I want to demonstrate in this paper is that we can retain this kind of ethical significance, this ineliminable and irreducible transcendence of the other, as seen in the other’s face, and still stand firmly on naturalistic grounds to gain an understanding of its significance. Although I view Levinas as presenting an important challenge to science and naturalistic philosophy, and in that regard I want to take this challenge seriously, my argument will not be in total agreement with Levinas. I’ll argue that the transcendence at stake in this context involves one’s capacity to perceive in the other the possibilities of further interactions that have the potential to take one beyond oneself. The transcendence isn’t just in the other; it’s in our possible interactions with the other.

## Interacting with faces

What is it about the other’s face, or more generally, about the other, that elicits the ethical response? I want to work this out in terms that relate to recent debates in social cognition—and specifically in the context of embodied and enactive cognition. In this regard, I will reject what may appear to be a rather easy solution—an easy way to explain the transcendence of the other. That is, I will reject the idea that what transcends the face is the mind—the mental states (intentions, emotions, etc.) that somehow may be physically expressed in facial expressions but are themselves truly hidden and relatively transcendent, behind or beyond physical expressions.

The idea that the mind is hidden away, and thus transcendent to embodied comportment has been called the *unobservability principle* (UP; Krueger, 2012, p. 149). Leslie (1987, p. 164) provides a clear statement of UP: “Because the mental states of others (and indeed ourselves) are completely hidden from the senses, they can only ever be inferred”. Many such statements of UP can be found in the theory of mind (ToM) literature. Karmiloff-Smith (1992, p. 138), for example, contends that ToM “involves inferences based on unobservables (mental states, such as belief)‥.” Or again, Johnson (2000, p. 22): “Mental states, and the minds that possess them, are necessarily unobservable constructs that must be inferred by observers rather than perceived directly”.

In opposition to ToM, however, phenomenologists have argued against the supposed ubiquity of mindreading by theoretical inference or simulation, and have defended an embodied/enactive view that social understanding depends, in large part, on interaction rather than on mindreading (Gallagher, 2001, 2005, 2012; Gallagher and Hutto, 2008; De Jaegher et al., 2010). In interactive contexts direct perception also plays an important role in social cognition (Gallagher, 2008); and one’s perception of the other is often focused on the face. The argument in favor of interaction theory (IT) has turned out to be a large, complex, and controversial one. I will not try to provide the entire story or enter into many of the details in this paper. Rather, with a focus on the role of face perception, I will discuss some of the experimental literature and its interpretations. Much of the interpretation that we find in this literature is consistent with UP and the ToM approach, and in this regard it follows a common explanatory principle, namely, that social cognition will ultimately be explained once we identify the process or mechanism within the individual agent responsible for the individual’s ability to understand the other. In contrast to this principle, and consistent with interactionist views on social cognition, I’ll argue that in basic (and most) instances social cognition is accomplished by something that goes beyond the individual agent, namely, the interaction itself. I’ll suggest that it is in this interaction that we will be able to find an explanation of the kind of transcendence discussed by Levinas in the ethical context.

My aim in this section is not to provide an exhaustive review of the empirical literature on face perception, but to cover some of the relevant research pertinent to a range of social-cognitive experience. A good starting point is the research of Meltzoff and Moore (1977, 1994) on neonate imitation. These well-known, but still controversial experiments show that infants from birth are typically able to interact with their caregivers, in a way that privileges the face. Most of the experiments are on the imitation of facial gestures, such as tongue protrusion, mouth opening, and pursing of lips. But there are also experiments that show the infant is able to imitate angular tongue protrusion, movement of eyebrows, as well as smiles, grimaces, frowns, and so on (e.g., Field et al., 1982). We can note that one important aspect of these findings is simply that infants are attracted to faces. To explain this basic fact, Meltzoff and Moore (1997) propose an explanation in terms of a *cross-modal* mechanism. Faces are attractive and meaningful to infants because what the infant sees is generally isomorphic to own felt experiences. The cross-modal integration of vision and proprioception allows the infant to make some kind of pragmatic sense of the other’s expression, in a way that calls forth a response (see Gallagher and Meltzoff, 1996), or as Meltzoff and Moore (1997) put it, it calls forth an action which serves the specific purpose that the infant is able to employ imitation to verify the identity of others.

There are disagreements about whether this kind of response is genuine imitation, or whether it’s the result of perceptual priming in a system with underdeveloped inhibitory mechanisms, or simply a form of contagion (see, e.g., Hurley and Chater, 2004). There are, accordingly, disagreements about the nature of the activity and the nature of the mechanisms to be found in the infant that would account for their ability. Without settling these kinds of disagreements, proponents of IT consider neonate imitation to be part of “primary intersubjectivity” (Trevarthen, 1979), and, regardless of how it is explained or what internal mechanism is involved, its significance is primarily that it is a very early process that pulls the infant into a dyadic and dynamic interaction with the other. One can set aside questions about whether the infant is conscious of what it is doing, or whether internal representations are involved, or whether it’s a strictly automatic response that comes down to mirror neuron activation, and still see that the significance of the infant’s response to the other’s face is tied to the fact that it is not a one-way process. The adult initiates the process in a way that elicits the infant’s response and establishes an interaction that is two-way or reciprocal. The infant comes to be enactively coupled to the other in this interaction. The idea of enactive coupling means, in this context, that (1) it is a dynamic process (i.e., one in which a co-dependence is established between the coupled systems such that what happens in or to one system is partly dependent on the situation of the other); (2) that the recurrent engagement with the other person leads to a structural congruence between self and other (Thompson, 2007, p. 45); and (3) that the engaging organisms (or agents) maintain their autonomy (their own internal self-organization).^1^ Accordingly, although one can still talk of individuals who engage in the interaction, a full account of neonate imitation is not reducible to mechanisms at work in either or both of the individuals. Complex coordination patterns that result from the mutual interaction of a social encounter, as such, are not simply inputs to individual mechanisms (De Jaegher et al., 2010). Such coordination processes can acquire a momentum of their own and can pull the participants into further or continuing interaction. Interaction in intersubjective contexts goes beyond each participant; it results in something (the creation of meaning) that goes beyond what each individual qua individual, that is, on its own, can bring to the process.^2^

Tracking and discriminating faces are some of the earliest infant capacities (Stern, 1985; Johnson et al., 1991; Walton et al., 1992; Hendricks-Jansen, 1996; Mondloch et al., 1999; Bushnell, 2001). Faces have saliency, not only for newborn infants, but throughout the life span, and many, if not most of our interactions with others are conducted face-to-face where enactive coupling is the rule, and where interaction itself is enabling and sometimes constitutive of social cognition. Developmental studies indicate the continued importance of faces. We know that infants “vocalize and gesture in a way that seems [affectively and temporally] ‘tuned’ to the vocalizations and gestures of the other person” (Gopnik and Meltzoff, 1997, p. 131) and that “in the gentle, immediate, affectionate, and rhythmically regulated playful exchanges of proto-conversation, 2-month-old infants look at the eyes and mouth of the person addressing them while listening to the voice” (Trevarthen and Aitken, 2001, p. 6). This has been dramatically demonstrated in still face experiments (Tronick et al., 1978) where the animation of the other’s face is shown to be absolutely essential to the interaction process. The advent of joint attention (sometime during the first year—see Reddy, 2008), “secondary intersubjectivity” (Trevarthen and Hubley, 1978), as well as social referencing (Klinnert et al., 1983; Mumme et al., 1996) all depend on making visual contact with the face of the other.

Eye-tracking studies and our everyday phenomenology attest to the fact that the importance of the perception of the other’s gaze for a grasp of intentions and emotions continues in adulthood. We see meaning and emotion in the faces of others. Phenomenologists have noted this often in their criticisms of the UP.

I do not see anger … as a psychic fact hidden behind the gesture …. The gesture does not make me think of anger, it is anger itself. … I perceive the grief or the anger of the other in his conduct, in the face or his hands, without recourse to any “inner experience” (Merleau-Ponty, 2002, pp. 214, 415).

Anger, shame, hate, and love are not psychic facts hidden at the bottom of another’s consciousness: they are types of behavior or styles of conduct which are visible from the outside. They exist on this face or in those gestures, not hidden behind them (Merleau-Ponty, 1964, pp. 52–53).

As Merleau-Ponty understands the notion of behavior, it is not a meaningless set of movements that require us to make inferences beyond what we can see. Behavior is meaningful, and what we can see is the meaning and the intention in the actions and expressions of others. Accordingly, this is not a form of psychological behaviorism. The phenomenologists are not alone in this. Consider that Wittgenstein, a philosopher from a very different tradition, says much the same thing.

Look into someone else’s face, and see the consciousness in it, and a particular shade of consciousness. You see on it, in it, joy, indifference, interest, excitement, torpor, and so on.… Do you look into yourself in order to recognize the fury in *his* face? (Wittgenstein, 1967, §229). [I]t is as if the human face were in a way translucent and that I were seeing it not in reflected light but rather in its own (Wittgenstein, 1980, §170).

In intersubjective contexts, visual perception of the face of the other is not equivalent to glancing at an object. It’s not a matter of me seeing the other’s face, *simpliciter*, but of seeing that the other sees me (or quiet literally, seeing the other seeing me). The fact that the other returns the gaze, and that this strongly registers in our perception (cf. Sartre, 1956; Stawarska, 2009), provides part of the basis for regarding the other not as mere object but as a perceiving subject—and carries with it ethical significance. The other’s gaze is precisely not something that can be subsumed into a strictly visual representation of eye direction since it has an affective impact on my own system that sets me up for further response. Perception of another’s face activates not just the face recognition area and ventral pathway, but also the dorsal visual pathway—suggesting that we perceive affordances for possible responsive actions in the face of the other (Debruille et al., 2012). Faced with the face of a real person, at a minimum, subjects make eye contact with very subtle eye movements. Accordingly, face perception presents not just objective patterns that we might recognize as emotions. It involves complex interactive behavioral and response patterns arising out of an active engagement with the other’s face—not a simple recognition of facial features—but an interactive perception that constitutes the recognition of emotions.^3^

It’s a mistake, of course, to take the face as an isolated entity, or to think that face-based emotion recognition is informationally encapsulated (pace Goldman, 2006, p. 110), even if in many cases we focus on the face in everyday life. We rely on a variety of bodily aspects in social interaction—posture, movement, gesture, vocal intonation and prosody—as well as communicative and narrative practices (Gallagher and Hutto, 2008), place-related and contextual factors, background knowledge about the person, etc. and our own prior experience. In this regard, we can also say that some of what is true of perception in general also applies to face perception. For example, meaningful perception of any sort may rely on activation of association brain areas outside of very early perceptual processing areas, like visual cortex V1. But recent research shows that even neuronal activity in the earliest of perceptual processing areas, such as V1, reflects more than simple feature detection. E.g., V1 neurons anticipate reward if they have been relevantly attuned by prior experience (Shuler and Bear, 2006). What we see in the present, including faces, incorporates an affective sense of relevant past experiences, so that reportable visual perception is already informed with affective value from the start. Barrett and Bar’s *affective prediction hypothesis* “implies that responses signaling an object’s salience, relevance or value do not occur as a separate step after the object is identified. Instead, affective responses support vision from the very moment that visual stimulation begins” (Barrett and Bar, 2009, p. 1325). Along with the earliest visual processing, the medial orbital frontal cortex is activated and initiates a train of muscular and hormonal changes throughout the body, “interoceptive sensations” from organs, muscles, and joints associated with prior experience, which are integrated with current exteroceptive sensory information that help to guide response and subsequent actions. Accordingly, along with the perception of the environment, we also undergo and possibly experience, more or less recessively, certain bodily affective changes that accompany this integrated processing (Barrett and Bar, 2009, p. 1326). In other words, before we fully recognize an object or a face, for what it is, our bodies are already configured into overall peripheral and autonomic patterns based on prior associations. In terms of the predictive coding model used by Barrett and Bar, priors are not just in the brain, but involve a whole body adjustment.

Disruptions to intersubjective interaction and to emotional attunement can equally enlighten us about the nature of social cognition. If a facial expression contradicts other interactive or communicative processes, for example, if an actor shows happy facial gestures while telling a sad story (Decety and Chaminade, 2003), the result is puzzlement, distrust, and more explicit attempts to figure out motivations. If perception of the emotion pattern on the face is disrupted, intersubjective problems develop.

While most people perceive the face or body of another as a familiar whole imbued with life, subjectivity, and expression, schizophrenia patients will sometimes focus on individual parts or the purely material aspect of the person before them (see Addington and Addington, 1998; Sass and Pienkos, 2013).

As a result, in such instances of schizophrenia (as well as in autism), subjects have a propensity to view the face as an array of unrelated details; they miss the pattern/gestalt and fail to recognize the emotion.

In cases of Möbius Syndrome (MS)—a form of congenital bilateral facial paralysis resulting from developmental problems with the sixth and seventh cranial nerves (Briegel, 2006)—subjects lack the capacity for facial expression and full control of eye movements. These physical problems can lead to difficulties with social understanding and behavior. Some subjects with MS manifest traits of social inhibition, introversion, feelings of social inadequacy and inferiority (Briegel, 2007) and report feeling out of sync with others (Cole, 1999b; Cole and Spalding, 2009). Indeed, part of the problem in MS is not *in* MS itself, but in the regard of others and in interactions between people with MS and others. Because facial expressions play a large role in intersubjective interaction, we anticipate facial responses and when they do not occur (as in MS) interaction can be disrupted in terms of its dynamics and affectivity, leading to confusion or feelings of social discomfort. This does not rule out the possibility that people with MS can find alternative strategies for interacting and understanding others (as Krueger and Michael, 2012 argue),^4^ but it does highlight the importance of facial expressions for social cognition.

Face-related problems with intersubjective interaction are also to be found in cases of blindness (both congenital and acquired), those on the autism spectrum who actively avoid looking at faces, those with facial disfigurements or Parkinson’s Disease. Jonathan Cole (1999a) gives an excellent account of these conditions with respect to the social difficulties that come along with them. Cole also takes us back to issues raised by Levinas.

If face-to-face relationships involve feelings toward and between people, any external face, another’s face, puts a demand on me. It asks me to recognize another, for what I cannot fully assimilate I must respect, and for Levinas this recognition summons me to a form of moral responsibility, in the face of the other, which cannot be brought under the control of my reason and therefore cannot be explained. This moral or ethical responsibility can be viewed in terms of the need for a response, for the face of the other requires me to respond and enter into a relationship, but a relationship that I cannot fully control, that neither of us can fully control. It involves a risk so evident for many of those with facial problems that they avoid it (Cole, 1999a, p. 196).

## About face: responding to Levinas

It’s clear from the various empirical studies cited above that, as Krueger and Michael (2012) so aptly put it, “the face is the center of gravity for our social interactions” Krueger and Michael (2012, p. 4). But there is also something that seemingly floats free of a purely physical science. Levinas insists on transcendence. For him, I experience transcendence “when the face has turned to me, in its very nakedness. It is by itself, and not by reference to a system” (Levinas, 1969, p. 75). Levinas associates war with the concept of totality (a complete system, the opposite of a never complete infinity) and a denial of morality: “War renders morality derisory” (Levinas, 1969, p. 21). In this regard it is notable that the face of the other in battle has profound inhibitory effects on violent behavior directed towards the other (Grossman, 1996; Protevi, 2008). Killing involves an objectification (or de-subjectification) of the other in practices that include covering or ignoring the other’s face. In this particular context, the denial of the face signifies that the other is reduced to a complete system which excludes the possibility of any further interaction. One finds this same denial, a closing down of interaction possibilities, in cases of torture and solitary confinement (Guenther, 2013; Gallagher, 2014).

I want to suggest, along with Levinas (1969), and adopting his terms, that “infinity is produced in the relationship of the same with the other” (Levinas, 1969, p. 26). But this means that there is nothing about the face in itself, *solus ipse*, or on its own, that generates the ethical demand. Nor is there anything like a complete alterity of the other that is not already mediated in interaction; much of what I am is already shaped in my interactions with others. Levinas emphasizes the asymmetrical demand of the other on me (e.g., Levinas, 1969, p. 46). Yet, we could think that the ethical demand is generated in the mutual turning towards each other. What is important is that the other looks back at me, as I meet her gaze with my own—this mutual experience, which is an aspect of primary intersubjectivity, sparks an interaction between me and the other. The transcendence associated with the ethical is not something unreachable in the other, but is generated in the interaction that transcends all individuality.

The most basic and primary experience of the other is this face-to-face, which sets in play the interaction and the transcendence—an interaction that transcends the individuals involved and requires a response that is never complete. The meaning that emerges or is established by the interaction calls for further interpretation, interaction or communication. The ethical, which is about our way of living with others, is built around this primary intersubjective experience—and around it we start to build certain practices.

Interactionists sometimes use the metaphor of the tango (e.g., Di Paolo et al., 2013). Just as it takes two to tango, one cannot accomplish interaction by oneself. Just as when two people dance the tango, something dynamic is created that neither one could create on one’s own. One might think that the metaphor of the tango involves an overly formal structure and that perhaps something more like a free dance form is more appropriate for how the dynamics of interaction work. But most of our lives are lived within social and intersubjective structures (practices and institutions) that do specify how we relate to one another. In some cases this takes the shape of a norm or rule that requires that we mutually recognize our responsibility to the other. Even within such structures, however, even in those that may support totalizing practices, but perhaps short of war, torture, and solitary confinement, one can find the possibility of transcendence in face-to-face relationships. In that interaction there is a mutual expectation of response, and an expectation that we will continue the interaction to some defined or perhaps ill-defined and imperfect end.

Levinas is right about the face, and about its irreducibility; but the other’s face is not an absolute alterity, nor does it lead us beyond what we can find in our daily interactions.

On the one hand, in this realm (and clearly in the realm of some institutions) there are no guarantees that we find the kind of transcendence that Levinas talks about. On the other hand, the transcendence that may be found in interactions can open up a vista of possibilities—possibilities of further interactions that have the potential to take me beyond myself, and that make the other incalculably significant, someone I turn away from at my own risk.

## Conflict of interest statement

The author declares that the research was conducted in the absence of any commercial or financial relationships that could be construed as a potential conflict of interest.

## Footnotes

^1^See Di Paolo and De Jaegher (2012) for a more formal account of dynamic coupling.

^2^As Di Paolo et al. (2008) put it, “interaction can dynamically create phenomena that do not directly result from the individual capacities or behaviors of any of the partners if investigated on their own” (p. 279).

^3^If we think of emotions as complex patterns of experiences and behaviors—and as such as “individuated in patterns of characteristic features”—features that may include bodily expressions, behaviors, action expressions, etc., then emotion perception can be considered a form of pattern recognition (Izard, 1972; Izard et al., 2000; Newen et al., in press). In this regard, the facial expressions play a major role.

^4^I’m in favor of a pluralist approach to social cognition (Fiebich, 2012), which does not deny that we can use some form of theoretical inference or simulation (see Gallagher, 2001), as well as narrative and communicative practices to gain understanding of others (Gallagher and Hutto, 2008). Pace Krueger and Michael (2012); I do not deny such possibilities. I do think, however, there is significant behavioral and phenomenological evidence to suggest that most of our everyday encounters with others are primarily embodied interactions, including communicative interactions, and that third-person theoretical inference and simulation are exceptional rather than common. I note also that I’m not at all convinced that a reverse simulation model as a form of mimicry can be thought of as “endorsing” what Krueger and Michael (2012) call strong interaction theory, as they claim.
